# Ohio District Dental Societies

**Published:** 1888-11

**Authors:** 


### Ohio District Dental Societies.

At the recent meeting of the Ohio State Dental Society the feasibility of organizing District Dental Societies, that should embrace the entire State was discussed at considerable length, and the unanimous opinion, so far as expressed, was that such organization should be effected, and that they should be so arranged territorially as to accommodate the whole profession of the State. Associative work has beyond any question been one of the greatest influences in developing, building-up and strengthening the profession in this country. Unfortunately, however, but a small proportion of the profession as a whole has as yet availed themselves of this means of improvement, and this movement looks to the securing of the interest and co-operation of all in this work.

The plan contemplates dividing the State into six or eight districts, in such a manner as to be most convenient to the members, and to assign to each district a proper and efficient quota of workers. There are already two societies of this character in the State, viz. : The Northern Ohio Society and the Mad River Society. These would not in any way be interfered with, except perhaps to modify the territory of each somewhat for the sake of uniformity and proper adjustment. The Mississippi Valley Society does not belong to Ohio any more than to half-a-dozen other States, and so it will in no wise be affected. Neither will the city societies be influenced by these organizations; they will be, and remain just what the profession of the cities chose to make them.

The question of making these district societies auxiliary to the State Society is an important one. It seems eminently proper, and, indeed, there are many reasons why they should be so organized: The fact of their being auxiliary to the State Society would serve as a common bond of union between the different societies; they would also derive strength from such an alliance, and in turn they would afford support and strength to the State Society, which would increase its efficiency for good.

With a view of giving aid and co-operation in this work, the State Society appointed a committee of five persons. This committee consists of Dr. L. P. Bethel, of Toledo; Dr. H. H. Harrison, Cadiz; Dr. C. R. Butler, Cleveland; Dr. J. Taft, Cincinnati; Dr. A. F. Emminger, Columbus.

This committee will doubtless be pleased to confer with members of the profession throughout the State in regard to the contemplated organizations, and give any aid and suggestions that may be in their power.

